# A Novel Kunitzin-Like Trypsin Inhibitor Isolated from Defensive Skin Secretion of *Odorrana versabilis*

**DOI:** 10.3390/biom9070254

**Published:** 2019-06-28

**Authors:** Yanjing Dong, Daning Shi, Yuan Ying, Xinping Xi, Xiaoling Chen, Lei Wang, Mei Zhou, Qinan Wu, Chengbang Ma, Tianbao Chen

**Affiliations:** 1 Natural Drug Discovery Group, School of Pharmacy, Queen’s University Belfast, Belfast BT9 7BL, UK; 2 School of Government, Peking University, No 114, The Leo KoGuan Building, Beijing 100871, China; 3 College of Pharmacy, Nanjing University of Chinese Medicine, Nanjing 210023, China

**Keywords:** frog skin secretion, trypsin inhibitor, Kunitz-type inhibitors, kunitzins

## Abstract

Protease inhibitors that were identified from amphibian skin secretions with low molecular weights and potent inhibitory activity were thought to be potential candidates for novel peptide drugs. Here, a novel peptide with trypsin inhibitory activity was found in the skin secretion of the Chinese bamboo leaf odorous frog, *Odorrana versabilis*. Based on the sequence alignments of sequencing results, the novel peptide (ALKYPFRCKAAFC) was named as Kunitzin-OV. The synthetic replicate of Kunitzin-OV was subjected to a series of functional assays, and it exhibited a trypsin inhibitory activity with a Ki value of 3.042 µM, whereas, when Lys-9 at P1 position was substituted by Phe, trypsin inhibitory activity was undetected and the chymotrypsin inhibitory activity was optimized with a Ki value of 2.874 µM. However, its protease-binding loop was catabolized by trypsin during the trypsin cleavage test. In conclusion, Kunizin-OV is a novel peptide with trypsin inhibitory activity as a member of kunitzins, which is a non-typical Kunitz-like trypsin inhibitor with a highly conserved reactive site (K-A) and quite a short sequence.

## 1. Introduction

Trypsin inhibitors (TIs), which belong to the serine peptidase inhibitors family, can competitively bind to trypsin via a substrate-like manner to regulate the action of trypsin [1]. These inhibitors are widespread in both plants [2] and animals [3,4], and have been described to possess great clinical significance in cancer prevention [5], treatment of obesity [6], and celiac disease therapy [7].

Skin secretions from amphibians are an abundant source of multi-functional bioactive peptides, and these peptides are useful agents for hydration, and regulating and strengthening defensive mechanisms [8]. Over the past decades, many different types of protease inhibitors have been identified from amphibian secretions, and these inhibitors with low molecular weights and potent inhibitory activity were thought to be potential candidates for novel peptide drugs [1]. According to the similarities of structural domains, sequences, reactive sites, and mechanisms, these inhibitors can be classified as different types (e.g., Kazal-, Bowman–Birk-, and Kunitz-type) [9]. For example, Kazal-type inhibitors have been isolated from phyllomedusine frogs [10], Bowman–Birk inhibitors from *Odorrrana* frogs [11], and Kunitz-type inhibitors from the tomato frogs [3] and ranid frogs [12]. Among these three frog skin-derived inhibitors, Kunitz-type has been the least studied.

Furthermore, Kunitz-type inhibitors are also known as bovine pancreatic trypsin inhibitor (BPTI)-like proteins. While BPTI is the classic member of this family of proteins and was the first Kunitz-type protease inhibitor described, it has relatively broad specificity inhibiting trypsin as well as chymotrypsin and elastase-like serine (pro) enzymes [13]. In general, Kunitz-type inhibitors consist of 50 to 60 amino acid residues and are stabilized by a disulphide bond-rich structure, and a highly exposed P1 active site residue for interacting with proteases (trypsin mostly) is usually arginine or lysine [13,14]. 

Odorous frogs are distributed in East Asia and the surrounding areas, and their skin secretions have been well studied with several trypsin inhibitors from these species having been reported [11,14,15]. As a member of the *Odorrana* genus, *Odorrana versabilis (O. versabilis)* is chosen as a research object, which has a great potential to provide novel and functional protease inhibitors. 

In this study, the cloning of skin-derived cDNAs and the identification and structural characterisation of a novel peptide with potent trypsin inhibitory activity are described. According to bioinformatic analysis, this peptide is a member of the Kunitz-type inhibitor family with a canonical Kunitz-type reactive centre. Meanwhile, a P1-substituted analogue is also synthesized and evaluated.

## 2. Materials and Methods

### 2.1. Specimen Biodata and Secretion Acquisition

Eight specimens of *O. versabilis* (6–8 cm snout-to-vent length, sex undetermined) were collected in the field in China. All frogs were kept in a vivarium at 25 °C under a 12 h/12 h day/night cycle and were fed crickets three times per week. The collection of skin secretion was performed as in the previous study [10], and the secretion was finally lyophilized and stored at −20 °C before analysis. This study was approved by the Nanjing University of Chinese Medicine Ethical Review Board—Approval Code: SYXK (SU) 2018-0048.

### 2.2. Molecular Cloning of Kunitzin-OV Precursor-Encoding cDNA from the Skin Secretion-Derived cDNA Library of O. versabilis

The lyophilized skin secretion was subjected to a series of procedures including mRNA isolation, cDNA library construction, cloning, and sequencing to obtain the biosynthetic precursor of Kunitzin-OV [10]. Specially, for Kunitzin-OV, 3′-RACE was facilitated by a nested universal primer (NUP) (supplied by the kit) and a sense primer (REry-3: 5′-GAWYYAYYHRAGCCYAAADATG-3′), which was designed to a highly conserved domain of the 5′-untranslated region of previously characterized antimicrobial peptide cDNAs from *Rana* species.

### 2.3. Identification and Structural Analysis of Kunitzin-OV from Skin Secretion of O. veserbilis

Lyophilized skin secretion was dissolved and then subjected to reverse-phase HPLC using a Waters gradient reverse phase high-performance liquid chromatography (HPLC) system as detailed in the previously published article [11]. The molecular masses of peptides in each fraction were further analysed by use of a matrix-assisted laser desorption ionization time-of-flight (MALDI-TOF) mass spectrometer (Voyager DE, PerSeptive Biosystems, Foster City, CA, USA), and selected fractions were then infused into an LCQ Fleet ion trap electrospray mass spectrometer (Thermo Fisher Scientific, San Francisco, CA, USA) followed by trapping of suitable ions for MS/MS fragmentation [11].

### 2.4. Solid-Phase Peptide Synthesis of Kunitzin-OV and F^9^-Kunitzin-OV

Kunitzin-OV and F^9^-Kunitzin-OV were synthesized by standard Fmoc chemistry using a Tribute™ peptide synthesizer (Protein Technologies, Tucson, AZ, USA) that was described in the previous study [16]. The crude peptides were purified and identified via the combination of HPLC and MS technology. The lyophollized pure peptides were subjected to a series of functional assays. The physicochemical properties of the peptides were computed using ProtParam. 

### 2.5. Minimal Inhibitory Concentration Assay of Kunitzin-OV and Phe-Substituted Analogue

*Staphylococcus aureus* (NCTC 10788), *Escherichia coli* (NCTC 10418), and *Candida albicans* (NCPF 1467) were applied in the antimicrobial test. They all were cultured in Mueller-Hinton Broth (MHB). The broth dilution method was applied to detect the minimal inhibitory concentrations (MICs) of peptides and the peptide concentrations were arranged from 1 μM to 512 μM in a two-fold dilution [16]. Peptides were incubated with subcultured bacteria cells (5 × 10^5^ cfu/mL) and each concentration was performed in seven replicates.

### 2.6. Haemolysis Assay

As described in the previous study [16], a suspension of horse red blood cells (4%, *v*/*v*) (TCS Biosciences, Botolph Claydon, Buckingham, UK) was incubated with peptides of the concentration range of 1 µM to 512 µM at 37 °C for 2 h to determine the peptide haemolytic activity. The positive control employed 1% TritonX-100 (Sigma, UK).

### 2.7. E. coli Cell Membrane Permeability Assay of Kunitzin-OV

Kunitzin-OV was prepared at its (the minimum, inhibitory concentration) MIC and then incubated with subcultured *E. coli* cells (1 × 10^8^ cfu/mL), after that, a SYTOX^TM^ green nucleic acid stain (Life Technologies, Paisley, UK) was applied for the determination of *E. coli* cell viability [17]. Moreover, the cell-penetrating peptide melittin (512 µM) was served as a positive control, while sodium phosphate-buffered saline (PBS) was served as a negative control.

### 2.8. Trypsin/Chymotrypsin Inhibition Assay

The inhibitory activity of peptides towards trypsin and chymotrypsin was tested the same as detailed in Wang’s study [11]. Peptides were initially prepared in a range of 10^−3^–10^−6^ M and then diluted to 16 concentrations. Each dilution was tested in duplicates. Additionally, Phe-Pro-Arg-AMC and Succinyl-Ala-Ala-Pro-Phe-NHMec (obtained from Bachem, UK) were served as substrates for trypsin and chymotrypsin, respectively.

### 2.9. Trypsin Cleavage of Kunitzin-OV

One milligram of synthetic Kunitzin-OV was subjected to the trypsin cleavage assay, following the method described in the previous study [12], with a minor difference. Briefly, the enzymatic reactions were stopped at 0, 1, 2, 5, 10, and 20 min, and every sample was dropped on the 100-well target plate and subjected to MALDI-TOF analysis.

## 3. Results

### 3.1. Molecular Cloning and Sequencing Analysis of Kunitzin-OV

**Figure 1:**
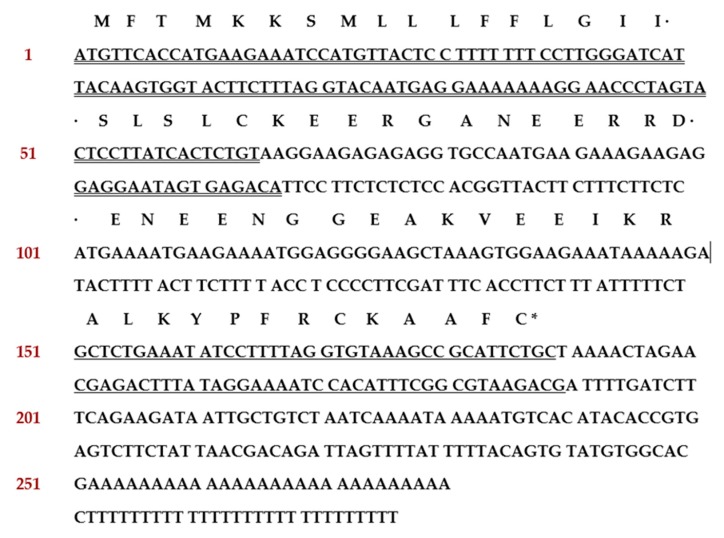
The open-reading frame of the biosynthetic precursor of Kunitzin-OV. The putative signal peptide is double-underlined, the mature peptide is single-underlined, and an asterisk indicates the stop codon.

**Figure 2:**
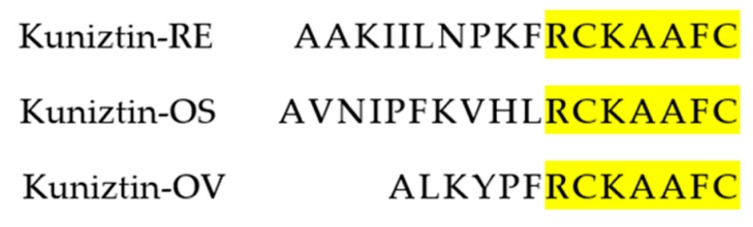
The primary structure of the novel Kunitzin-OV from *Odorrana versabilis* skin secretion compared with other kunitzins. Fully conserved residues are highlighted.

From the skin-derived cDNA library of *Odorrana versabilis*, the cDNA encoding the biosynthetic precursor of a putative novel bioactive peptide named Kunitzin-OV was consistently and repeatedly cloned. The open-reading frame of this cloned precursor consisted of 63 amino acid residues, which included a 22 amino acid residues signal peptide and a mature peptide of 13 amino residues. The putative peptide sequence was preceded by two successive basic amino acids, Lysine (K) and Arginine (R), which represented a typical cleavage site. A single disulphide bond (Cysteine 8-3) was located at the C-terminal (Figure 1). As the Basic Local Alignment Search Tool (NCBI-BLAST) analysis and sequence alignments showed, this novel peptide shares an identical C-terminal part with previously found kunitzins (Figure 2). The biosynthetic precursor encoding cDNA of Kunitzin-OV was deposited into GenBank with an accession number of MK965543. 

### 3.2. Identification and Structural Characterisation of Kunitzin-OV

**Figure 3:**
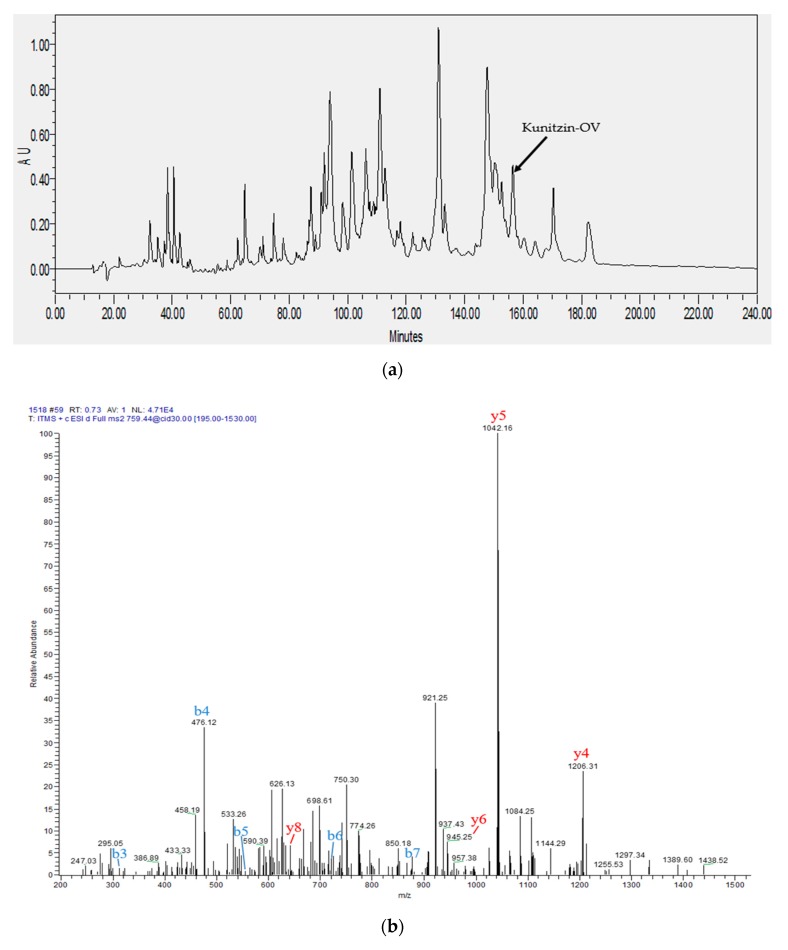
The identification and characterisation of Kunitzin-OV: (**a**) the 240 min reverse-phase high-performance liquid chromatography (HPLC) chromatogram of the skin secretion of *Odorrana versabilis*. The elution position is indicated with an arrow. The components were monitored at a wavelength of 214 nm; (**b**) peptide fragment spectrum collected on an LCQ Fleet ion trap mass spectrometer. The peptide was fragmented randomly at each peptide bond, b- and y-ions are indicated in blue and red.

After a 240 min gradient reverse-phase high-performance liquid chromatography (HPLC) analysis, each fraction was identified by matrix-assisted laser desorption ionization time-of-flight mass spectrometry (MALDI-TOF MS). After comparison of the computed and the determined molecular masses of Kunitzin-OV, the elution site of the peptide was confirmed, while the peptide structure was characterized by tandem mass spectrometry (MS/MS) fragmentation sequencing (Figure 3).

### 3.3. Synthesis of Kunitzin-OV and Phe-Substituted Variant

The F^9^-Kunitzin-OV and Kunitzin-OV were synthesized from C-terminus to N-terminus along with the sequences by solid-phase peptide synthesis (SPPS). According to the data computed by ProtParam, the modified F^9^-Kunitzin-OV possessed less positive charge, but a higher grand average of hydropathicity than the original peptide does (Table 1).

### 3.4. Antimicrobial/Haemolytic Assay of Kunitzin-OV and F^9^-Substituted Analogue

**Figure 4:**
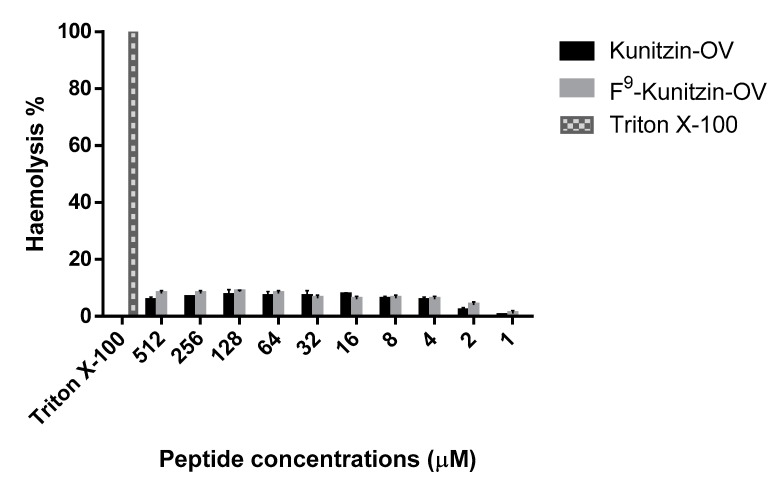
The haemolytic activity of Kunitzin-OV and F^9^-Kunitzin-OV against horse blood cells. Triton X-100 was severed as a positive control with 100% haemolysis.

No significant antimicrobial activities were observed for Kunitzin-OV and its F^9^-substituted analogue (Table 2). Both peptides showed little haemolytic activity (less than 10%) even at concentrations up to 512 μM (Figure 4).

### 3.5. E. coli Cell Membrane Permeability of Kunitzin-OV

**Figure 5:**
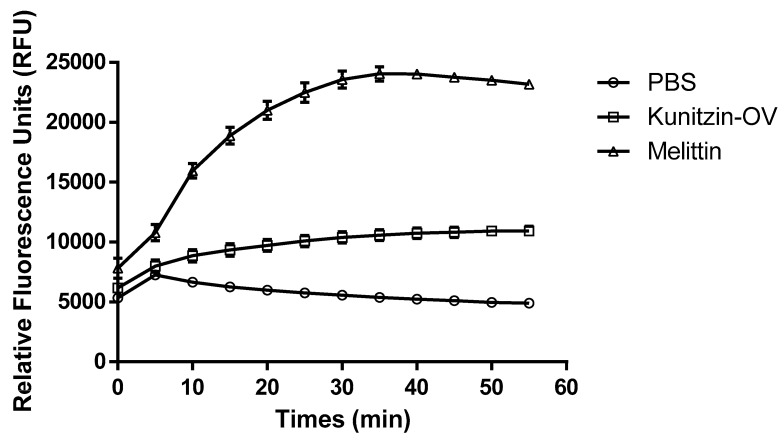
The cell membrane permeability of Kunitzin-OV with phosphate-buffered saline (PBS) as a negative control and melittin as a positive control.

The cell membrane permeabilization activity of Kunitzin-OV was tested with its MIC value (512 µM), as shown in Figure 5. Compared to the cell-penetrating melittin, Kunitzin-OV only exhibited a low degree of permeable effect on the *E. coli* cell membrane.

### 3.6. Trypsin/Chymotrypsin Inhibitory Activity of Kunizin-OV and Phe-Substituted Analogue

**Figure 6:**
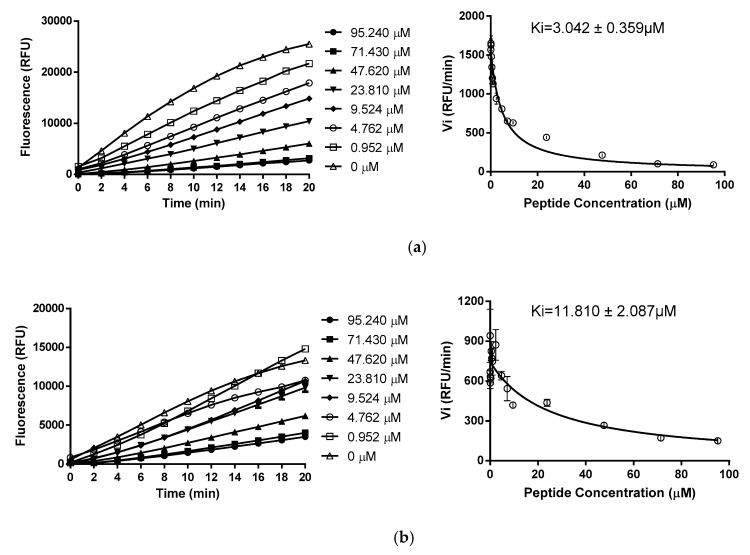

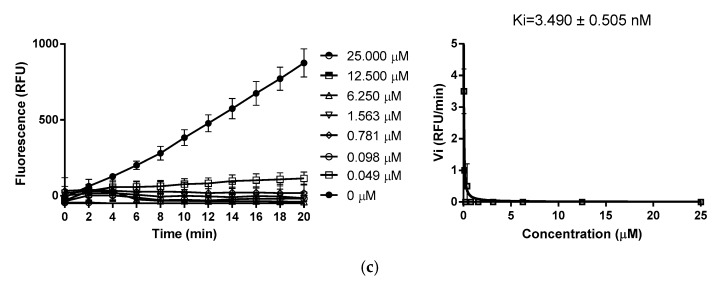
Trypsin/chymotrypsin inhibitory activities: (**a**) Reactive curves and corresponding Morrison plot of trypsin inhibition of Kunitzin-OV in various concentrations; (**b**) Reactive curves and corresponding Morrison plot of chymotrypsin inhibition of F^9^-Kunitzin-OV in various concentrations; (**c**) Reactive curves and corresponding Morrison plot of trypsin inhibition of ovomucoid inhibitor (chicken) in various concentrations.

**Figure 7:**
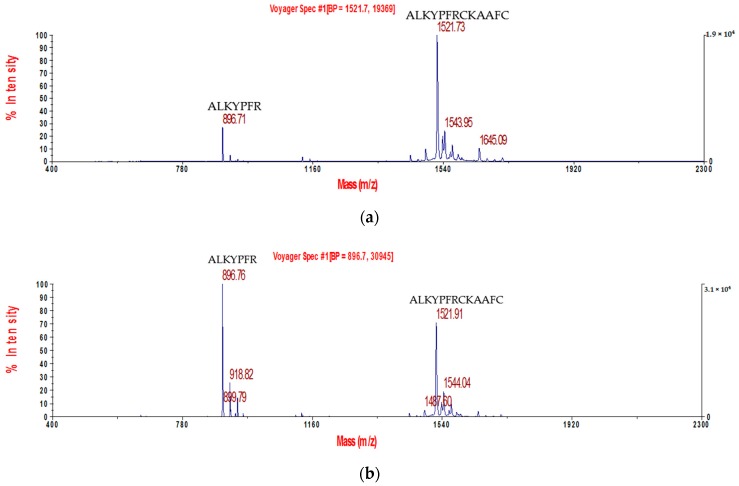

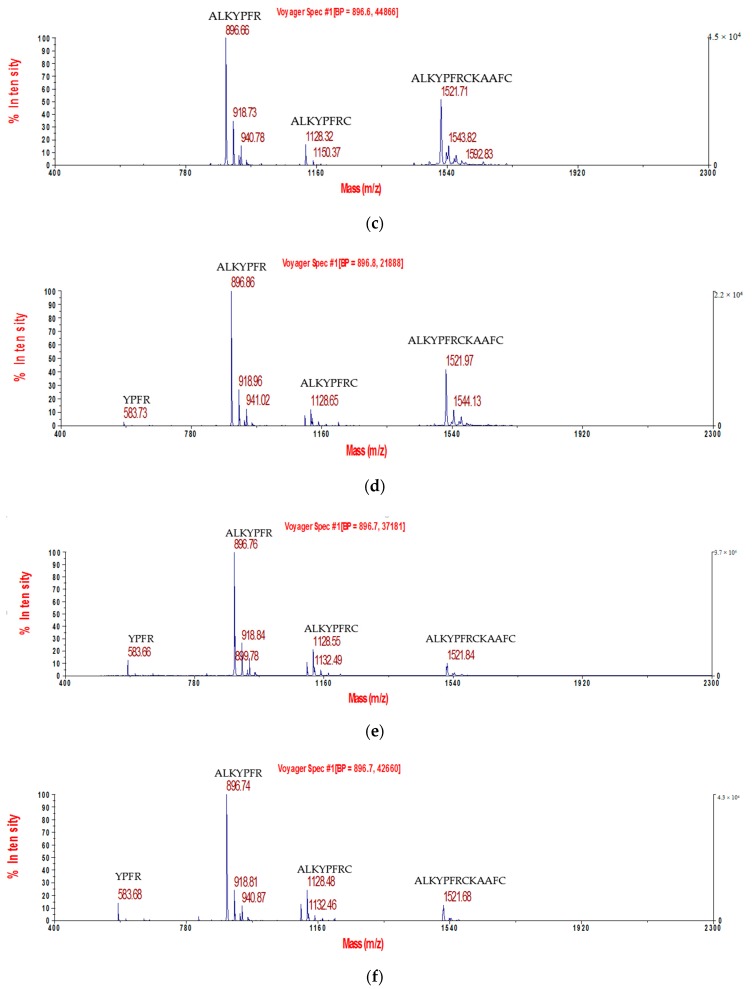
The trypsin cleavage results obtained by matrix-assisted laser desorption ionization time-of-flight (MALDI-TOF) analysis for Kunitzin-OV from different time points, (**a**–**f**): 0 min, 1 min, 2 min, 5 min, 10 min, and 20 min. The obtained fragments are marked in the figure.

Kunitzin-OV displayed a trypsin inhibitory activity with a Ki value of 3.042 ± 0.359 µM. With a Lys-9 residue occupying the P1 position, no chymotrypsin activity was observed. Whereas, when the Lys-9 at P1 position was substituted by a Phe, trypsin inhibitory activity disappeared, and chymotrypsin optimized inhibitory activity was obtained, with a Ki value of 11.810 ± 2.087 µM. Besides Ki values, progress curves and Morrison plots of Kunitzin-OV and its variant are also shown (Figure 6). The chicken egg white trypsin inhibitor Type III-O, which is also called ovomucoid inhibitor, was served as a contrast, and it showed faster and stronger inhibitory efficacy on trypsin than Kunitzin-OV (Figure 7).

### 3.7. Trypsin Cleavage Assay of Kunitzin-OV

The synthetic replicate of Kunitzin-OV was subjected to trypsin cleavage assay. It can be seen from the MALDI-TOF results that Kunitzin-OV was cleaved into small catabolites along with the time (Figure 7). The obtained fragments were marked in the figure, which shows that the catabolites were cleaved through typical trypsin cleavage sites.

## 4. Discussion

Odorous frogs are distributed in East Asia and the surrounding areas, and they are named because of their smelly skin secretion, which plays a fundamental role in defensive systems [18]. To date, hundreds of functional peptides have been isolated from *Odorrana* skin secretion, not only many potent antimicrobial peptides but also trypsin inhibitors. For instance, a small trypsin inhibitor OGTI was identified from *Odorrana grahami* (with a Ki value of 0.4 µM) in 2008 [14], and a potent Bowman–Birk-type trypsin inhibitor (with a Ki value of 388 ± 28 nM) was initially isolated from the skin of the Hejiang odorous frog, *Odorrana hejiangensis* [11]. Additionally, a new class of protease inhibitor with trypsin inhibition (with a Ki of 5.56 mM) was reported in the skin secretion of *Odorrana schmackeri* [12].

In this study, a novel peptide was isolated from the skin secretion of the Chinese bamboo leaf odorous frog, *Odorrana versabilis*. It is a short peptide composed of 13 amino acid residues, which contain a single disulfide bond (-CKAAFC-) located at the C-terminal between Cys^8^ and Cys^13^. The primary sequence of this novel peptide was subjected to homology searches using NCBI-BLAST, and its C-terminal part was 100% identical to the corresponding sequence of a kunitzin precursor. The name kunitzin was initially reported for two trypsin inhibitors, Kunitzin-RE and Kunitzin-OS, from *Rana esculenta* and *Odorrana schmackeri,* respectively [12]. Based on the comparison of structure homology, the six-residue disulfide bond of the novel peptide is identical to the C-terminal loop of the previously reported kunitzins, while their amino acid residues are arranged in a different order at the N-terminal. Besides, the novel peptide in this study was determined to possess the ability to inhibit trypsin as kunitzins do.

In the antimicrobial assay, Kunitzin-OV exhibited no significant inhibition against either bacteria or yeast, which was quite different from the results obtained from the test on Kunitzin-RE (MIC *E. coli* = 30 µM) [12], as this reported peptide possessed a narrow-spectrum antimicrobial activity against Gram-negative bacterium *E. coli*. The results just corresponded to the physicochemical properties of these two peptides. Kunitzin-RE contains one more net charge (+4) and a higher GRAVY index (0.567) than Kunitzin-OV, in which a more positive charge brings better electrostatic attraction between a cationic peptide and an anionic bacteria membrane surface, and higher hydrophobicity promotes the peptide interaction with the hydrophobic tail of bacteria bilayer [19]. Moreover, these results also suggested that the disulfide bond (-CKAAFC-) is not a decisive factor for antimicrobial activity, as Chen [12] and his colleagues also synthesized this single loop alone to test its antimicrobial properties, and no activity was observed against all tested microbes. In 1995, Sonohara and his partners [20] investigated the differences between the surface of *S. aureus* and *E. coli* via electrophoretic mobility measurements, and they found that *E. coli* had a more negatively charged and less soft surface than that of *S. aureus*. Meanwhile, *C. albicans* is a potentially pathogenic yeast. It is a eukaryotic organism and has different cell wall components than bacteria [21]. These may explain why Kunitzin-RE only showed limited activity against *E. coli*.

As we known, melittin is a potent antimicrobial peptide (AMP) with broad-spectrum activities, besides it is also one of famous membrane-penetrating AMPs that is able to induce pore formation of the bacteria cell membrane [22]. It is clear that Kunitzin-OV exhibited much lower potency than melittin to permeabilize the cell membrane of *E. coli*, which could explain the weak antimicrobial activity of Kunitzin-OV.

In a further investigation, Kunitzin-OV was subjected to protease inhibitor assay, and it showed inhibition of trypsin with a Ki value of 3.042 µM and no inhibitory activity towards chymotrypsin. Based on the bioinformatics analysis, the C-terminal disulphide-bridged loop of Kunitzin-OV displayed a high degree of similarity to the reactive centre of several Kunitz-type inhibitors from different sources (Table 3). The P1 position of all these inhibitors is occupied by either a Lys or an Arg residue. Additionally, a study of the evolutionary trace about 74 Kunitz sequences has appeared that the P4 position of those Kunitz proteins is completely conserved as a glycine residue when partitioning identity cut-offs at high values [23]. However, Kunitzin-OV contains the conserved P1 residue without a Gly at the P4 position. Additionally, including the other two kuniztins mentioned above, OGTI [14] is also a trypsin inhibitor (AVNIPFKVHFRCKAAFC) from *Odorrana grahami* that shows the identical reaction loop but the absence of a Gly at P4.

Furthermore, the Ishikawain-2 from *Odorrana ishikawae*, which also contains the C-terminal six-residue loop, was first considered as an antimicrobial peptide, but no antimicrobial activity was found after testing [20]. We thought it was highly likely to be a member of this particular kind of trypsin inhibitor, but surely further research is needed to prove it. Therefore, we considered that these inhibitors from amphibian skin secretions were a form of short and unique inhibition motif for possessing the self-defence efficiently. 

By replacing Lys-9 with Phe, the site-substituted analogue showed an optimized chymotrypsin inhibitory activity, while the inhibitory activity towards trypsin was eliminated. Generally, Kunitz-type inhibitors achieve their inhibition through a standard mechanism (canonical inhibition). They interact with the target via a tight and non-covalent bond, which is just like the enzyme-substrate complex [13]. Without any changes of conformation, the inhibitors block the active site of the serine protease, and an antiparallel β-sheet is formed between the enzyme and its inhibitor [24]. The so-called protease-binding loop refers to an extended, solvent-exposed and convex fragment that has charge of protease inhibitory activity, and the location of the reactive site (P1-P’1 peptide bond) is in the most visible part of this loop [1]. Crossing from P3 to P’3 position, the protease-binding loop is highly complementary to the concave enzyme active site [1]. 

As known, the catalysis of serine protease is specified by their S1 pocket, for trypsin, its negative charged Asp^189^-based S1 pocket selectively contacts with basic residues. Whilst, the S1 position of chymotrypsin, which contains phenyl group inside the pocket, shows higher affinity to aromatic residues [25]. Thus, in this study, -RCKAAF- is the trypsin-bonding loop and the reactive site (K-A) is complementary to the trypsin active site. Once the Lys-9 (P1 position) was substituted by Phe, the trypsin inhibitory activity vanished. It also suggested that the variant contains the reactive site (K-F) which works for the inhibition of chymotrypsin. It is a natural selection that a Lys residue occupied the P1 position of kunitzins to regulate the inhibitory activity towards trypsin.

In the case of Kunitzin-RE, the result is slightly different. Weak inhibition of trypsin (Ki = 48.37 μM) was still observed for the substituted analogue [12]. Combined with the data obtained from trypsin cleavage, each segment of both Kunitzin-OV and Kunitzin-RE was cleaved by trypsin via typical cleavage site Lys and Arg. The difference was the C-terminal function loop of Kunitzin-OV was catabolized via Lys-9, while this did not happen to Kunitzin-RE. Therefore, we assumed that the Lys close to the region of the protease-binding loop could act as a competitive position for trypsin activity and protect the functional disulphide loop from cleavage. In this way, the inhibitory activity may be more stable and productive. It has been described that association energy can be influenced by residues around the protease-binding loop and even from a distant region, and these residues can still contact the enzyme [4]. 

Based on the catabolites obtained in the trypsin cleavage assay, Kunitzin-OV was catabolized by trypsin through the classical cleavage sites Lys and Arg. These data may imply that Kunitzin-OV achieves its inhibition by competing with the substrate for the active site of the trypsin. Besides, the reactive site (K-A) was not cleaved at the very end of the incubation, which means this sequence was catabolized in a highly ordered way and the inhibitory reaction would continue until the reactive site was cleaved. Nevertheless, the reaction loop (-RCKAAF-) was cleaved via Lys-9 residue after 2 min of incubation, which might result in an unstable structure and a less potent activity of Kunitzin-OV. This may also explain why the ovomucoid inhibitor (chicken) showed more potent inhibitory activity than Kunitzin-OV did, as the ovomucoid consists of more than 200 amino acid residues that could stabilize the conformation. It might improve the affinity to the reaction centre of trypsin and slow down the degradation of the inhibitor. As a consequence, the short sequence of Kunitzin-OV was degraded by trypsin easily. Interestingly, it has been proved that inhibitors with a short sequence showed higher thermostability than ovomuciod inhibitors did [26], which implies that to increase the structural stability and keep the short sequence at the same time is an efficient way for the small peptide inhibitor to optimize the inhibitory activity.

As mentioned above, the addition of a Lys or Arg residue around the loop may be an efficient way to protect the P1 position from cleavage and thus improve the peptide stability and efficacy. Further research needs to be done to prove this. The study of Kunitzin-OV may propose a new insight for trypsin inhibitor-related therapeutics because they have been regarded as potential therapeutic molecules in the treatment of chronic diseases, like obesity [6]. 

## 5. Conclusions

In summary, Kunitzin-OV is a novel trypsin inhibitor isolated from the defensive skin of the odorous frog, *Odorrana versabilis.* It is considered as a member of kunitzins, which is a non-typical Kunitz-like trypsin inhibitor with a highly conserved reactive site (K-A) and quite a short sequence. This study of Kunitzin-OV provided a new option for the design of trypsin inhibitors. Moreover, the frog skin-derived kunitzins, which exhibited a short sequence, potent trypsin inhibitory activities, and little cytotoxicity, have great potential to be natural drugs.

## Figures and Tables

**Table 1:** Sequences and physicochemical properties of Kunitzin-OV and its Phe-substituted variant.

	Sequence	Net Charge	GRAVY ^1^
Kunitzin-OV	ALKYPFRCKAAFC	+3	0.354
F^9^-Kunitzin-OV	ALKYPFRCFAAFC	+2	0.869

^1^ GRAVY—the grand average of hydropathicity.

**Table 2:** Minimum inhibitory concentrations (MICs) determined for the Gram-positive bacterium, *Staphylococcus aureus (S. aureus)*, the Gram-negative bacterium, *Escherichia coli (E. coli)*, and the yeast, *Candida albicans (C. albicans)* with Kunitzin-OV and Kunitzin-OV variant. The blank control was established by the culture medium, and the positive control represented growth culture.

Peptides	Sequence	Minimum Inhibitory Concentration (µM)		
		*S. aureus*	*E. coli*	*C. albicans*
Kunitzin-OV	ALKYPFRCKAAFC	512	512	512
F^9^-Kunitzin-OV	ALKYPFRCFAAFC	512	512	512

**Table 3:** Comparison of the reactive sites of Kunitzin-OV with different kinds of Kunitz-type protease inhibitors. The reactive sites are highlighted.

Name	Sequence Length	Reactive Site	Source
Kunitzin-OV	13	-F R C K A A F-	This study
VKTO1_HAPHA	55	-G R C K A S F-	UniProtKB-D2Y2Q6
VKTCT_OPHHA	58	-G F C K A Y I-	UniProtKB-B6RLX2
BPT1_BOVIN	58	-G P C K A R I-	UniProtKB-P00974
CSTI_BOMMO	55	-G P C K G S F-	UniProtKB-P81902
SPIT2_HUMAN domain 2	51	-G P C R A S F-	UniProtKB-O43291
AMBP_HUMAN domain 2	51	-G P C R A F I-	UniProtKB-P02760
VKT_OXYSC	51	-G P C R A A I-	UniProtKB-B7S4N9
